# General Medicine and Therapeutics

**Published:** 1894-11

**Authors:** 


### GENERAL MEDICINE AND THERAPEUTICS.

EDITED BY DR. LUTHER B. GRANDY.

### THE HYPODERMIC ADMINISTRATION OF MERCURY.

In the *Journal des Practiciens*, August 11, 1894, Dr. Ch. Mauriac formulates the following rules:

All irritant or caustic preparations are, of course, inadmissible. The liquid injected must be chemically pure and sterile. The syringe must be sterilized in all its parts, and an unoxidizable needle (such as iridized platinum), not less than 1½ inches long, must be used. All injections must be given deeply into the tissues, the needle being inserted perpendicularly to the surface.

The lumbar portion of the back and the gluteal region are the best positions. Care must be taken not to introduce the syringe into a vein. Subsequent injection ought to be 2—2½ inches distinct from a previous one.

The interval between each injection varies with the effect produced and the preparation employed—from twice a day with small quantities of soluble solutions, to once a month with large injections of insoluble preparations, such as calomel.

Solutions employed :

(1) *Soluble Injections.* Corrosive sublimate.

| | | |
|---|---|---|
| Corrosive sublimate | 1 | part. |
| Chloride of sodium | | parts. |
| Sterilized water | 100 | " |

A syringeful (about 15 minims) to be injected every day.

To avoid such frequent injections, M. Lukesiewicz injects half to one syringeful of a stronger solution every four or eight days. Corrosive sublimate can also be dissolved in sterilized oil, so that 15 minims contain one-sixth of a grain, which is the usual dose.

Biniodide of mercury is also well borne, and causes little pain. One-sixteenth of a grain may be injected, dissolved in 15 minims of sterilized oil, and repeated every day or every other day, as the case may require.

Sozoiodolate of mercury is very active, but causes much pain. The following is Schwimmer's formula :

| | | |
|---|---|---|
| Sozoiodolate of mercury | 8 | parts. |
| Iodide of potassium | 14 | " |
| Distilled water | 100 | " |

About 6 minims are injected every week, and the dose may be carefully increased.

(2) *Insoluble Injections.* Calomel.

| | | |
|---|---|---|
| Sublimated calomel | 1 | part. |
| Liquid vaseline | 10 | parts. |

Fifteen minims to be injected every fifteen days.

Yellow oxide may be given in the same dose and manner as calomel.

Grey oil is less active than calomel.

One and a-half to three minims are injected every eight days.

| | | |
|---|---|---|
| Mercury (purified) | 20 | parts. |
| Etherial tincture of benzoin | 5 | " |
| Liquid vaseline | 40 | " |

Thymolate of mercury, 1 part, mixed with ten parts of liquid vaseline, and 7—15 minims given every eight days.

Salicylate of mercury may be used like the thymolate.

—*Medical Chronicle.*

---

### THE ANTITOXIN TREATMENT OF DIPHTHERIA.

H. Urquhart Walker, L.R.C.P. and S. Ed., late Medical Officer of Health, Worksop, Notts, writes: I was called in to see M. J., a girl, aged eight, on July 27th. I was told she had been ill for a day or two. She had passed a very restless night, and had been quite delirious. In the morning she had complained of her throat. Her temperature was 102° and her pulse 130. On examination, the tonsils and fauces generally red and swollen, and on the right tonsil was a patch of membrane, but not very large. On the 28th the breathing was more difficult; the throat presented much the same appearance as on the previous day. On the 29th the breathing was worse, and the fauces on the right and left sides were covered with membrane. In the evening the respiration was very quick. On the 30th the membrane was well formed, even extending well over the soft palate and hanging down into the mouth. Up to this time the child had been treated with iron and chlorate of potash internally, and the constant use of the lime-water spray. It was evident that something more must be done, so I resorted to antitoxin, a supply of which I had procured on reading Dr. Goodhart's case in the *British Medical Journal* of July 28th. Eleven minims of antitoxin were injected into the forearm with strict antiseptic precautions. The temperature at the time of injection was 103° and the pulse 140. Four hours after the injection the child was much improved. There was no fever, and the pulse was quite quiet. She had a good night, and had to be roused by the nurse to receive her nourishment. On the 31st the same improvement continued, although the membrane was still present, although loosening on the right side. The temperature was normal. During the day much of the membrane came away. The last patch to form had disappeared. All that was to be seen was on the uvula and on the left side. On August 1st all signs of membrane had disappeared. From that date uninterrupted recovery took place. I wish to call attention to the fact that within a few hours after using the antitoxin, the temperature was reduced 4°, and remained normal. The constitutional disturbance was at once checked, although the membrane still remained on the uvula and left side of the throat. The new patch on the soft palate at once disappeared, and the appearance generally became more normal. The injection was followed neither by local nor by general disturbance.—*British Medical Journal.*

---

### HOW TO COMPUTE THE DOSE FOR CHILDREN.

Dr. P. Bolognini contends that the formulæ in general use for computing the doses of remedies suitable for different ages are unsatisfactory. He has computed the average weight of children for each month of the first year, and for each year thereafter up to the eighteenth, and upon this basis has formulated the following rules:

1. From birth to the end of the first year: let $d$ stand for dose and $m$ for the age of the infant in months. Then the fraction of the adult dose will be represented by $d = \frac{1}{20 - m}$.

2. For a child from two to eighteen years of age the formula is $d = \frac{2 + a}{25}$; $d =$ dose, and $a =$ age of the child in years.—*Archives of Pediatrics.*

---

### NEW TEST FOR SALOL.

The *Pharmacuetishe Zeitschrift für Russland* recommends the following: If a few drops of nitro-sulphuric acid are added to a small quantity of salol in a china saucer, the mixture will first turn yellow, soon changing to brown, and finally to green. By adding water and stirring it becomes reddish, but ammonia will bring back the green color. Resorcin, similarly treated, takes on a beautiful dark-blue tinge, which water changes to red, but which is restored by ammonia.—*Drug. Cir. & Chem. Gaz.*

### INFANT FEEDING.

Percy Boulton, in the *British Medical Journal*, publishes a useful table showing the normal weight for height of children born at full term. The height and weight is given at six months periods, from birth up to the fifth year.

The table shows that an infant should double its weight in six months and treble it in a year if its nutrition is in every way satisfactory. The practical point is this: If a child does not increase at the rate of one pound a month during the first year of life, and twelve ounces a month during the second year, its nutrition is not satisfactory. If a child does not grow nearly three-quarters of an inch every month during the first year of life, and one-half inch a month during the second year of life, it is not satisfactory. The latter is, of course, not of the same importance as the former. Clearly premature children would not be so large, though they should increase at the same ratio.—*Canadian Practitioner.*

---

### THE STRYCHNINE TREATMENT OF PULMONARY CONSUMPTION.

Dr. Thos. J. Mays, in the *College and Clinical Record* says that, next to rest and food, strychnine in large doses is the most important agent in the treatment of pulmonary consumption. Begin with $\frac{1}{32}$ of a grain, and gradually increase to $\frac{1}{16}$, $\frac{1}{10}$, or $\frac{1}{6}$ of a grain, or even larger doses, given four times a day. According to the author, it does not produce albuminuria or diabetes, as is generally supposed. It alleviates the loss of appetite, the vomiting, the constipation, the nervousness and sleeplessness, the pain in the chest, the cough and expectoration, the dyspnœa, the weakness of the heart, and acts as a blood-builder in an eminent degree. Its usefulness rests, of course, on its influence over the nervous system, and is another link in the chain of evidence which shows that in the great majority of cases pulmonary consumption is the direct result of primary disease of the pulmonary nerve supply.

---

THE following is a broad rule: Dropsy of the feet alone means heart, dropsy of the belly alone means liver; and dropsy of all the body means kidneys.—*Exchange.*

### INHALATION FOR NASAL, PHARYNGEAL, AND BRONCHIAL CATARRH.

In considering the treatment of this condition, Dr. Clarence Rice (*Archives of Pediatrics*, April, 1894) recommends the following formula:

| | |
|---|---|
| Menthol | grs. v. |
| Thymol | grs. v. |
| Carbolic acid | grs. v. |
| Oil of eucalyptus | ℨ ii. |
| Oil of pinus sylvestris | ℨ iii. |

Directions: A teaspoonful on the boiling water, or twenty or thirty drops on the sponge or absorbent cotton. Inhale for ten or fifteen minutes.

The spout of the croup kettle can be placed near the head of the patient while she is asleep, and both the child and kettle inclosed under a sheet. This warm medicated steam will liquefy the secretions, diminish congestion, relieve the dyspnœa, and stop the cough. Nicely finished apparatus for giving these inhalations may be found in the market, though they are on the principle of the croup kettle, which can be manufactured cheaply in any tin shop.—*Canadian Practitioner.*

---

### SOME PRESCRIPTIONS.

*Cracked Nipples.*—M. Lepage uses compresses soaked in the following solution:

| | | |
|---|---|---|
| ℞ | Red iodide o mercury | gr. iss–iij. |
| | Alcohol | ℨ iss. |
| | Distilled water | ℨ xivss. |
| | Glycerin | ℨ xvj. |

M.—*Universal Medical Journal.*

*Dysentery in Children.*—When the pain and straining are intense, relief may be derived from the following:

| | | |
|---|---|---|
| ℞ | Cocaine, muriat | gr. j. |
| | Ext. ergot., aq | gr. x. |
| | Ext. opii, aq | gr. ij. |
| | Aristol | gr. v. |
| | Ol. theobrom | q. s. |

M. ft. suppos. no. x.

Sig.: One every two or three hours.—*Prescription.*

*Migraine.*—Freudenberg prescribes:

℞ Hydrochlorate of morphine........................gr. 1-6.
Salicylate of sodium..............................gr. iv.
Phenacetin........................................gr. iv.

M. One or two such cachets according to need.

Or, pastilles composed of:

℞ Saccharin.........................................gr. 1-6.
Hydrochlorate of quinine..........................gr. 5-6.
Salicylate of sodium..............................gr. iiss.

M. One pastille at a dose.—*Le Progrès Méd.*

*Chlorosis from Menorrhagia.*—

℞ Sulphate of iron..................................gr. c.
Ext. hydrastis candensis..........................gr. c.
Ext. hyoscyamus...................................gr. 1.

Divide into one hundred pills; two to be taken at each meal.—*Med. Press and Cir.*

*Antiseptic Powder for Cancer of the Uterus.*—Luataud insufflates the following powder daily, the os being exposed by means of a speculum:

℞ Acid. salicyl.....................................gr. iv.
Acid. boric.......................................℥ i.
Iodoformi.........................................ʒ ii.
Ess. eucalypt. q. s.

—*Der Frauenarzt.*

*Neuralgia.*—For stubborn neuralgia try the following:

℞ Antipyrin.........................................ʒ iss.

Caffeine..........................................ʒ ss.

Ext. cannabis ind.,

Ext. aconite, aa..................................gr. iiss.

Hyoscyami hydrobromat.............................gr. ⅓.

M. et ft. caps, no. xxx.

Sig.: One every two or three hours.—*Prescription.*

*Suppositories for Hemorrhoids.*—

℞ Aristol...........................................ʒ i.
Extract of opium..................................gr. ii.
Extract of belladonna.............................gr. ii.
Muriate of quinine................................gr. xv.
Cacoa butter.

White wax, of each, a sufficiency to make six suppositories.—*Prov. Med. Journal.*

